# Comprehensive Analysis of Prognostic Value of MEX3A and Its Relationship with Immune Infiltrates in Ovarian Cancer

**DOI:** 10.1155/2021/5574176

**Published:** 2021-06-03

**Authors:** Panpan Zhang, Tong Su, Shu Zhang

**Affiliations:** Department of Gynecology and Obstetrics, Shanghai Key Laboratory of Gynecology Oncology, Renji Hospital, Shanghai Jiao Tong University School of Medicine, Shanghai 200127, China

## Abstract

MEX3A is a critical RNA-binding ubiquitin ligase that is upregulated in various types of cancer. However, the correlations of MEX3A with prognosis and its molecular mechanism in ovarian cancer (OC) remain unclear. The expression level, prognostic values, and the genetic variations of MEX3A were analyzed via Gene Expression Profiling Interactive Analysis (GEPIA) Oncomine, Kaplan–Meier plotter, and cBioPortal. We used the LinkedOmics database to investigate the functions of MEX3A coexpressed genes and performed visualizing gene interaction network analysis on the GeneMANIA website. The correlations between MEX3A and cancer immune infiltration were analyzed by the Tumor Immune Estimation Resource (TIMER) site and the TISIDB database. Furthermore, *in vitro* analysis was performed to evaluate the biological functions of MEX3A in OC cells. Our study showed that the expression of the MEX3A in OC was higher than in normal tissues; it had the greatest prognostic value in OC, and strong physical interaction with PABPC1, LAMTOR2, KHDRBS2, and IGF2BP2, which indicated the association between MEX3A and immune infiltration. We also found that MEX3A was negatively related to infiltrating levels of several types of immune cells, including macrophages, neutrophils, dendritic cells (DCs), B cells, and CD8+ T cells. Additionally, *in vitro* experiments demonstrated that MEX3A promotes proliferation and migration in OC cells. Taken together, MEX3A might influence the biological functions of OC cells by regulating the immune infiltration in the microenvironment as a prognostic biomarker and a potential therapeutic target.

## 1. Introduction

Ovarian cancer (OC) is a common gynecological malignancy with high mortality. More than 70% of patients with OC are diagnosed with advanced-stage cancer (III and IV) [1]. Although the development of surgery and chemotherapy in ovarian cancer has been advanced in recent decades, the benefits of traditional treatment are limited [2]. Recently, immunotherapy has offered a novel and promising therapeutic strategy. Still, immunotherapy, which has been developing rapidly resulting in major breakthroughs in many areas, cannot achieve a good treatment effect because of a special tumor immune microenvironment [3]. Like many other solid tumors, OC is immunogenic, and the imbalance between immune activation and immune suppression can lead to tumorigenesis and cancer progression. Thus, it is necessary to select and identify reliable immune-related biomarkers and novel targets for immunotherapy strategies necessary to diagnose OC early.

MEX3A is an important component of the Mex3 family, which has a conserved region of about 70 amino acids, including MEX3A, MEX3B, MEX3C, and MEX3D [4]. MEX3A is a kind of RNA-binding protein (RBPs), which has the highly conserved RNA-binding domain and a C-terminal RING finger domain that are involved in posttranscriptional regulatory mechanisms [5]. Recently, MEX3A has been reported as a novel biomarker promoting proliferation and migration in various cancers such as pancreatic ductal adenocarcinoma (PDA), liver cancer, and colorectal cancer [6–8]; yet, its role in OC is still unclear.

In this study, we investigated the mRNA expression, mutation patterns, and prognosis value of MEX3A in OC for the first time based on large database analyses including Oncomine, GEPIA, cBioPortal, PrognoScan, and the Kaplan–Meier plotter. We also explored the function of the coexpression genes with MEX3A to clarify the potential mechanism in OC by GO and KEGG. In addition, we revealed the potential relationship between the expression of MEX3A and immune infiltration in the OC microenvironment via TIMER and TISIDB. We have further demonstrated that MEX3A enhanced tumor proliferation and migration in vitro. Collectively, our findings revealed the important role of MEX3A and provided a novel target and a valuable insight into the underlying mechanism between MEX3A and tumor-immune interactions in OC.

## 2. Materials and Methods

### 2.1. Oncomine Analysis

The MEX3A mRNA expression level was analyzed in OC by the Oncomine platform (http://www.oncomine.org/), a publicly accessible, online cancer microarray database with 715 data sets and 86,733 samples that allow for a powerful genome-wide expression analysis [9]. We selected a*P*value of 0.01 and a fold change of 2 as the threshold, and ranked genes in the top 10% as significant.

### 2.2. GEPIA Analysis

Gene Expression Profiling Interactive Analysis (GEPIA) is an interactive web used to analyze the RNA sequencing expression, including The Cancer Genome Atlas (TCGA) tumor sample information and Genotype-Tissue Expression (GTEx) normal sample information. GEPIA provides a series of key interactive and customizable functions by using a standard processing pipeline (http://gepia.cancer-pku.cn) [10].

### 2.3. cBioPortal Analysis

The cBioPortal for Cancer Genomics (http://cbioportal.org) provides an online resource to explore, visualize, and analyze complex cancer genomics and clinical profile data from TCGA [11]. In this study, the cBioPortal was used to access genetic variations in MEX3A (amplifications, deep deletions, and missense mutations), DNA copy number alterations, and mRNA expression *z*-scores (RNA Seq V2 RSEM). The tab OncoPrint shows an overview of genetic alterations for each sample in MEX3A. Besides, coexpression datasets were analyzed according to the online instructions of cBioPortal, and the R package was used for further enrichment analysis.

### 2.4. LinkedOmics

LinkedOmics (http://www.linkedomics.orglogin.php) is a publicly available web tool used to provide multiomics data of 32 TCGA cancer types [12]. We used the linkInterpreter module to derive biological insights into coexpressed gene enrichment by using Pearson's correlation coefficient. These genes were presented in volcano plots and heat maps.

### 2.5. Functional Enrichment Analysis

To further explore the functions of MEX3A, Gene Ontology (GO) enrichment analysis and Kyoto Encyclopedia of Genes and Genomes (KEGG) enrichment analysis were performed in R statistical computing environment.

### 2.6. GeneMANIA

GeneMANIA (http://www.genemania.org) is a flexible, friendly web interface that is used for visualizing gene interaction networks and evaluating gene function [13]. It enables analysis of gene lists and prioritizes the marked genes for functional assays associated with MEX3A. The sources of the edge of the network, which represent the following bioinformatics methods, namely, physical interaction, coexpression, colocation, genetic interaction, and website prediction, were set.

### 2.7. TIMER Database Analysis

To obtain the MEX3A expression and correlation between MEX3A and immunity cells in TCGA datasets, an online analytical tool called “Tumor Immune Estimation Resource (TIMER)” was used. TIMER is an online dataset used for evaluating the relationship between clinical associations, mutation, SCNA, and infiltration of different immune cells (B cells, CD4+ T cells, CD8+ T cells, neutrophils, macrophages, and dendritic cells) in diverse cancer types [14]. The survival module also showed the Kaplan–Meier plotter and provided the multivariable Cox regression analysis of clinical factors (age, stage, and tumor purity). Once all conditions were defined, TIMER outputs revealed the Cox regression results, including hazard ratios (HR), 95% confidence intervals (CI), and statistical significance (*P* < 0.05) automatically.

### 2.8. TISIDB Analysis

TISIDB (http://cis.hku.hk/TISIDB) is a user-friendly web portal, which contains a summary of 988 immune-related antitumor genes for 30 TCGA cancer types [15]. The associations between gene expression and immune features, including lymphocytes, immunomodulators, subtypes, and chemokines, were calculated by high-throughput data analysis. In this research, we used the TISIDB web to analyze the correlations between MEX3A expression and clinical stages, lymphocytes, and subtype immunomodulators in OC.

### 2.9. Kaplan–Meier Plotter Analysis

The Kaplan–Meier plotter (http://www.kmplot.com) is a common tool for biomarkers used to assess survival and prognosis, which includes gene expression data and survival information of 1,816 clinical tissue samples from OC patients [16]. The overall survival (OS) and progression-free survival (PFS) of OC patients were determined by dividing two groups (high vs. low expression) of patients by median. In addition, we further investigated OS and PFS of different histological subtypes (endometrioid and serous) in MEX3A by using the Kaplan–Meier method. These data were evaluated with a hazard ratio (HR), 95% confidence intervals (CI), and log-rank *P* value.

### 2.10. PrognoScan Database Analysis

The relationship between MEX3A expression and prognosis in OC was analyzed by the PrognoScan database (http://www.abren.net/PrognoScan/), such as OS and PFS [17]. The threshold was adjusted to a Cox *P* value < 0.05 or corrected *P* value < 0.5.

### 2.11. Cell Culture and Transfection

The ES2 cells were obtained from the Type Culture Collection of the Chinese Academy of Sciences (Shanghai, China) and cultured in Dulbecco's Modified Eagle's Medium (DMEM; HyClone; GE Healthcare Life Sciences) with 10% FBS at 37°C and 5% CO_2_. ES2 cells (to 70% confluence) were seeded on 6-well plates transfected with MEX3A siRNAs designed and synthesized by GenePharma (Shanghai, China), which were transfected into the cells using Lipofectamine 2000 (Invitrogen; Thermo Fisher Scientific) according to the protocols for the interference expression of MEX3A; the cells were cultured for 48 h or 72 h for further assays.

### 2.12. Cell Counting Kit-8 (CCK-8) Assay and Colony Formation Assays

Transfected cells were seeded on a 96-well plate at a density of 2 × 10^3^ cells/well. The CCK-8 solution (10 *μ*l; Dojindo Laboratories, Kumamoto, Japan) was then added to each well of the plate. The plate was incubated for 2 h in the incubator, and the absorbance at each wavelength of 450 nm was measured using an automatic enzyme-linked immune detector.

For the colony formation assay, transfected cells were seeded into 6-well plates. One week later, the cells were fixed with 4% paraformaldehyde and stained with 0.5% (*w*/*v*) crystal violet. Then, cell clones were photographed and counted. These experiments were performed in triplicate.

### 2.13. 5-Ethynyl-2-Deoxyuridine (EdU) Staining Assay

Collected cells were seeded on 24-well plates at a density of 1 × 10^4^ cells/well and incubated for 24 h. According to the protocol of the EdU Kit (BeyoClick™ EDU Cell Proliferation Kit with Alexa Fluor 488; Beyotime, Shanghai, China), after transfection, EdU was added 1 : 1,000 in the cell medium for 2 h at 37°C. Cells were fixed with 4% paraformaldehyde for 15 min and treated with 0.3% Triton-X for 10 min at room temperature. Then, the cells were incubated for 30 min with a Click reaction cocktail in the dark. Nuclei were stained with Hoechst 33342 for 10 min. Photographs were taken in three randomly selected fields with an Olympus (Tokyo, Japan) microscope to analyze proliferation rates. Each experiment was performed at least three times.

### 2.14. Transwell Assay and Wound Healing Assay

Cells (4 × 10^4^ cells/well) were incubated in 100 *μ*l culture medium and seeded on the Transwell inserts (Corning Glass Works; Corning, NY, USA) with 8 *μ*m pores to determine the migration ability of the cells. A 600 *μ*l culture medium was added to the lower chamber. After 48 h, the inserts were fixed with 95% ethanol, and 0.5% (*w*/*v*) crystal violet was used for staining. Migrated cells were counted in five nonoverlapping locations.

To analyze wound healing, we seeded transfected cells on 6-well plates. When the cell density reached 80-100%, we scraped cells at the bottom of the wells using a sterile 200 *μ*l pipette tip to form a linear gap and culture treated cells with FBS-free DMEM. After 24 h, images of the wells were taken with an inverted fluorescence microscope. All assays were repeated at least three times.

### 2.15. Quantitative Real-Time PCR

Total RNA was extracted using the TRIzol Reagent (Invitrogen; Thermo Fisher Scientific). According to the manufacturer's instructions, the concentration of total RNA was measured using Thermo Fisher Scientific NanoDrop ND-100. cDNA was synthesized using the SYBR PrimeScript RT-PCR Kit (Takara Bio, Inc., Japan). Real-time PCR was carried out using a Thermal Cycler Dice™ Real-Time system Tp800 (Takara Bio, Inc.). The primer sequences designed for MEX3A and *β*-actin are as follows (5′-3′): MEX3A, forward, TGGAGAACTAGGATGTTTCGGG, and reverse, GAGGCAGAGTTGATCGAGAGC; and *β*-actin, forward, CATGTACGTTGCTATCCAGGC, and reverse, CTCCTTAATGTCACGCACGAT. The mRNA expression of the target gene was analyzed using the 2^−ΔΔCt^ method.

### 2.16. Western Blotting

Total protein was obtained from cells using ice-cold RIPA buffer mixed with protease inhibitor cocktails (Roche), and concentration was assayed by a BCA assay. Fifty micrograms of denatured protein was separated by 10% SDS-PAGE and transferred onto PVDF membranes. After blocking with 5% skimmed milk for 1 h at room temperature, the membranes were incubated with antibodies against MEX3A (1 : 1000; ab79046; Abcam) overnight at 4°C, followed by incubation with a secondary antibody (1 : 3,000; #A0208; Beyotime, Beijing, China) at room temperature for 1 h. The ECL detection kit was used to detect protein signals.

### 2.17. Immunohistochemical (IHC) Staining

MEX3A expression was assessed by IHC assay, using previously described protocol [18]. Anti-MEX3A antibody (ab79046; Abcam) was used at a 1 : 50 dilution at 4°C overnight. Rabbit immunoglobulin G (1 : 1000; ab6721; Abcam) was used as a negative control. Aperio Scanning System (Aperio Group, LLC) was employed to scan the slides, and Aperio Image Scope software (version 10.2.2.2317, Aperio Technologies) was used for further quantitative analysis.

### 2.18. Statistical Analyses

Survival analysis was analyzed using the Kaplan–Meier method. GO enrichment analysis and KEGG enrichment analysis were performed under an R computing environment. Statistical analyses were performed using GraphPad Prism 7.0 (GraphPad Software, La Jolla, CA, USA). Comparisons were performed by a two-tailed Student's *t*-test. *P* values < 0.05 were considered statistically significant. Data were expressed as mean ± standard deviation (SD).

## 3. Result

### 3.1. High Expression Level and Prognostic Value of MEX3A in Ovarian Cancer by Bioinformatics Analyses

Firstly, to determine differences in MEX3A expression in tumor and normal tissues, the MEX3A mRNA levels in different tumors and normal tissues of various cancer types were analyzed using the Oncomine database. The database, which had a total of 241 unique samples for MEX3A, and a total of 22 cancers, including brain and CNS cancer, breast cancer, colorectal cancer, and ovarian cancer, showed that MEX3A mRNA levels were significantly upregulated in various cancers, and MEX3A expression in OC was high on top 5 (Figure S1a). GEPIA analysis also revealed similar results (Figure S1b).

**Figure 1:**
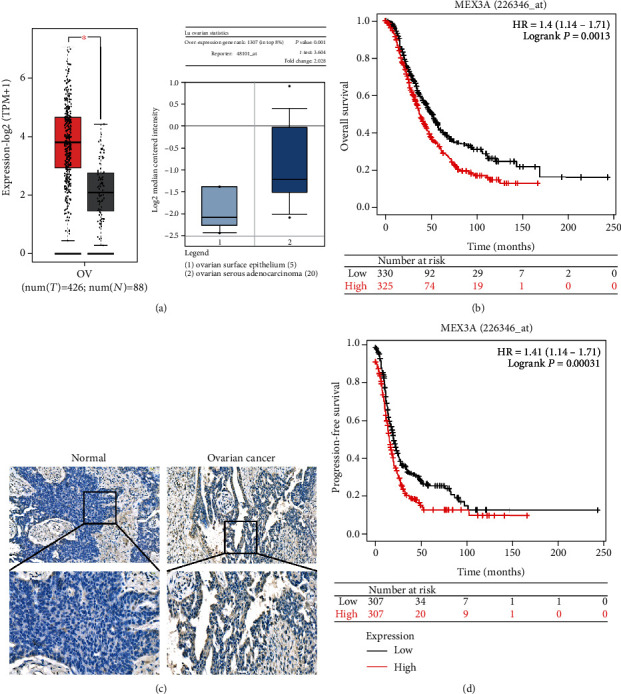
The mRNA expression levels and prognosis of MEX3A in ovarian cancer and normal tissues. (a) Box plots show mRNA expression of MEX3A in OC tissue (red plot) and normal tissues (gray plot) from GEPIA. (b) Prognostic significance of MEX3A in OC with OS from the Kaplan–Meier plotter. (c) Representative IHC images of MEX3A expression in normal tissue and ovarian cancer. (d) Prognostic significance of MEX3A in OC with PFS from the Kaplan–Meier plotter. The red survival curve represents high MEX3A expression, and the black survival curve represents low MEX3A expression in OC. ^∗^*P* < 0.05, ^∗∗^*P* < 0.01, and ^∗∗∗^*P* < 0.001.

Next, we found that MEX3A expression in OC significantly increased between 426 cases of OC and 88 cases of normal ovarian tissues via GEPIA (Figure 1(a)). In order to clarify the results, the expression differences in OC tissues (40 samples from Renji Hospital) and normal ovarian tissues (25 samples from Renji Hospital) were also validated by IHC staining (Figure 1(c)).

In addition, we investigated whether MEX3A was associated with prognosis in OC patients by using the Kaplan–Meier plotter and PrognoScan. The Kaplan–Meier plotter and PrognoScan databases showed that OC patients with high MEX3A expression experienced poor OS and PFS (Figures 1(b) and 1(d), Table 1). In order to explore the prognostic value of different histologies, the database revealed that higher MEX3A expression was correlated with shorter OS and PFS both in patients with endometrioid and serous cancers (Figures S1c–e). Collectively, MEX3A can be considered as an independent prognostic biomarker linked to a poor survival rate in OC.

### 3.2. MEX3A Expression Is Correlated with Immune Infiltration Level in OC

**Figure 2:**
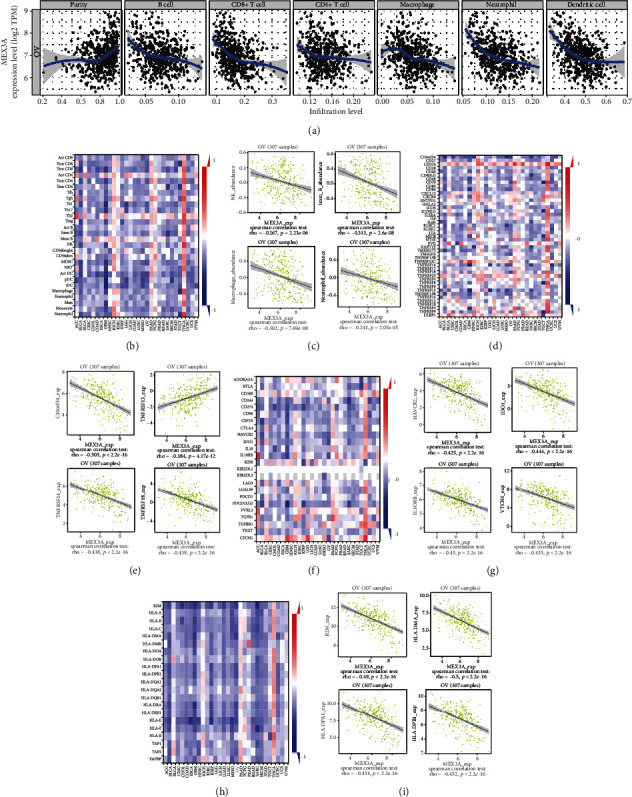
Correlation of MEX3A expression with immune infiltration level in OC. (a) Correlation of MEX3A expression with immune infiltration level in OC (TIMER). Spearman's correlation of MEX3A with lymphocytes and immunomodulators (TISIDB). (b) Heat maps of correlations between MEX3A expression and TILs by TISIDB. (c) Scatterplots of correlations between MEX3A expression and top 4 TILs. (d) Heat maps of correlations between MEX3A expression and immunostimulators. (e) Scatterplots of correlations between MEX3A expression and top 4 immunostimulators. (f) Heat maps of correlations between MEX3A expression and immunoinhibitors. (g) Scatterplots of correlations between MEX3A expression and top 4 immunoinhibitors. (h) Heat maps of correlations between MEX3A expression and MHC molecules. (i) Scatterplots of correlations between MEX3A expression and top 4 MHC molecules. Red and blue represent positive and negative correlations. The color intensity is directly proportional to the strength of the correlations. ^∗^*P* < 0.05, ^∗∗^*P* < 0.01, and ^∗∗∗^*P* < 0.001.

**Figure 3:**
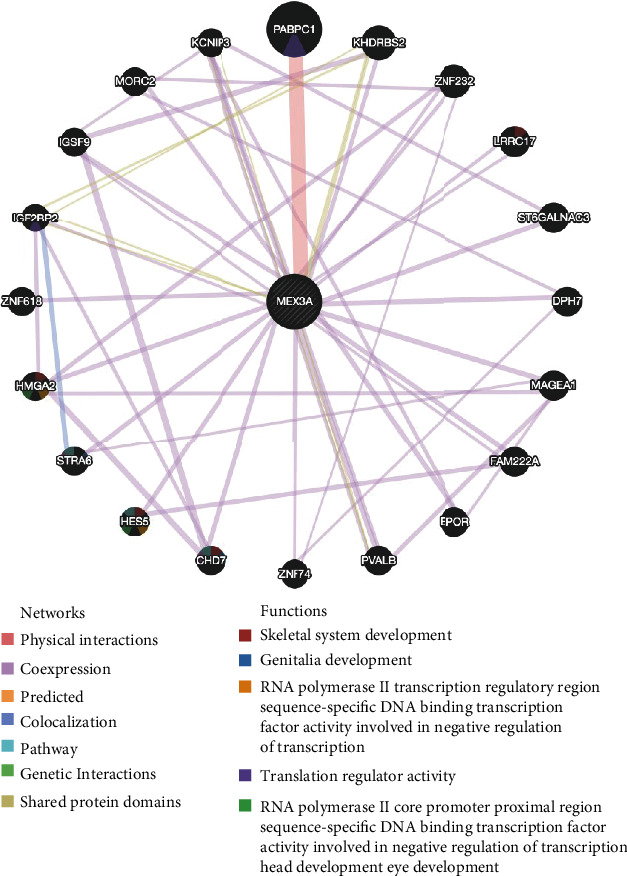
Gene-gene interaction network between MEX3A and correlated genes (GeneMANIA). Each node represents a gene. The node size indicates the strength of the interaction. The connecting lines between nodes represent the type of gene-gene interaction, and the line color represents the type of interaction. Node colors represent the possible biological functions of each gene.

To better understand the underlying mechanism of MEX3A in OC, we further investigated the relationships between MEX3A and the immune system. Tumor-infiltrating immune cells (TIICs) are an important part of the tumor microenvironment and which are independent predictors of cancer survival. It is unclear whether targeting MEX3A could influence the recruitment numbers of TIICs to impact the prognosis of cancers. Through TIMER analysis, we found that most immune cells were negatively correlated with MEX3A expression (Figure 2(a)). MEX3A expression had a negative correlation with B cells, CD8+ T cells, neutrophils and dendritic cells (DCs), and macrophages. However, the expression of MEX3A had weak associations with CD4+ T cells in OC. Subsequently, we used the TISIDB database to further analyze the relationship between MEX3A expression and immune regulation. Figures 2(b) and 2(c) show the correlation between MEX3A expression and TILs, which corresponded to the results reported above. Immunomodulators can be further divided into immunoinhibitors, immunostimulators, and major histocompatibility complex (MHC) molecules. Furthermore, we assessed the correlation between MEX3A expression and diverse immunomodulators. Figures 2(d) and 2(e) indicate the correlations between MEX3A expression and immunostimulators, and the greatest correlations include C10orf54 (Spearman's: *ρ* = −0.505, *P* < 2.2*e* − 16), TNFRSF18 (Spearman's: *ρ* = −0.439, *P* < 2.2*e* − 16), TNFRSF14 (Spearman's: *ρ* = −0.438, *P* < 2.2*e* − 16), and TNFRSF13C (Spearman's: *ρ* = 0.384, *P* < 2.2*e* − 16). Figures 2(f) and 2(g) indicate correlations between MEX3A levels and immunoinhibitors, where the strongest include IL10RB (Spearman's: *ρ* = −0.45, *P* < 2.2*e* − 16), IDO1 (Spearman's: *ρ* = −0.444, *P* < 2.2*e* − 16), VTCN1 (Spearman's: *ρ* = −0.435, *P* < 2.2*e* − 16), and HAVCR2 (Spearman's: *ρ* = −0.425, *P* < 2.2*e* − 16). Correlations between MEX3A expression and MHC molecules were also explored, and the greatest correlations include B2M (Spearman's: *ρ* = −0.49, *P* < 2.2*e* − 16), HLA-DMA (Spearman's: *ρ* = −0.5, *P* < 2.2*e* − 16), HLA-DPA1 (Spearman's: *ρ* = −0.451, *P* < 2.2*e* − 16), and HLA-DPB1 (Spearman's: *ρ* = −0.452, *P* < 2.2*e* − 16) (Figures 3(h) and 3(i)). Therefore, MEX3A may be involved in negative immune regulation.

### 3.3. Enrichment Analysis of Coexpression Genes Correlated with MEX3A in OC

**Figure 4:**
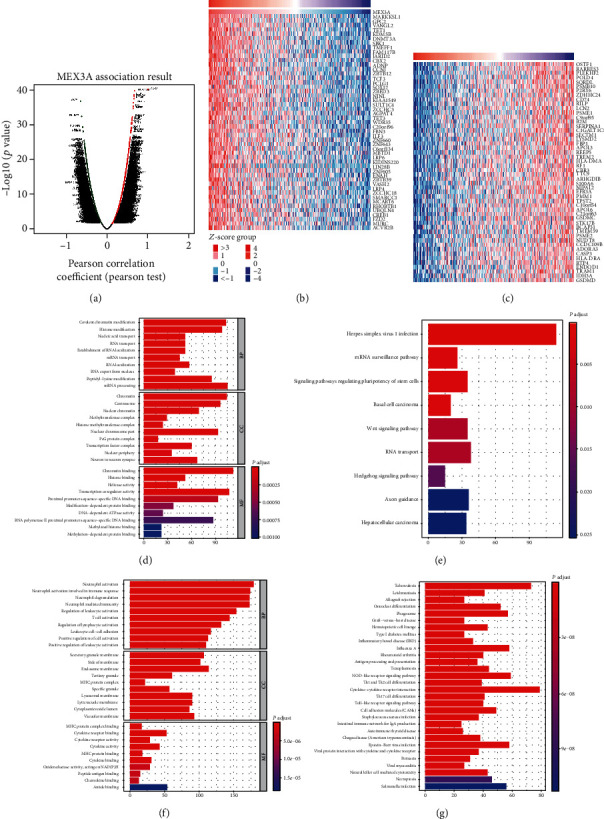
Genes differentially expressed in correlation with MEX3A (LinkedOmics). (a) Correlations between MEX3A and genes differentially expressed in OC. (b) Heat maps showing genes positively and negatively correlated with MEX3A in OC (Top 50). (c) Heat maps showing genes negatively correlated with MEX3A in OC. Red indicates positively correlated genes, and blue indicates negatively correlated genes. (d) Barplot representing enriched functions of the upregulated genes coexpressed with MEX3A. (e) Barplot representing enriched pathways of the upregulated genes coexpressed with MEX3A. (f) Barplot representing enriched functions of the downregulated genes coexpressed with MEX3A. (g) Barplot representing enriched pathways of the downregulated genes coexpressed with MEX3A.

Next, we analyzed mRNA sequencing data from OC patients in TCGA by using the function module of LinkedOmics. As shown in the volcano plot (Figure 2(a)), 2596 genes (dark red dots) showed significant positive correlations with MEX3A, and 3050 genes (dark green dots) showed significant negative correlations (FDR < 0.01). The 50 significant gene sets (such as ACTBL2, C12orf43, CCDC56, CCL27, CPNE8, FAM78B, KCTD17, and LAMB4) positively and negatively correlated with MEX3A are shown in the heat map (Figures 2(b) and 2(c)). These results indicated an important influence of MEX3A on the transcriptome level. Besides, GO term analysis showed that high expressed genes in correlation with MEX3A were mainly located in the chromatin centrosome and nuclear chromosome part, where they mostly participated in mRNA processing, covalent chromatin modification, and histone modification. Poor expressed genes were mainly located in the endosome membrane, secretory granule membrane, and side of the membrane and were involved in immune-related processing, including neutrophil and T cell activation and regulation of lymphocyte activation (Figures 4(d) and 4(e)). KEGG pathway analysis showed the most important enrichment in the herpes simplex virus 1 infection of high expressed genes and cytokine-cytokine receptor interaction of poor expressed genes (Figures 4(f) and 4(g)). These data pointed out that MEX3A might promote tumor progression by regulating immune cell response in the tumor microenvironment.

### 3.4. Genomic Alterations of MEX3A in OC

**Figure 5:**
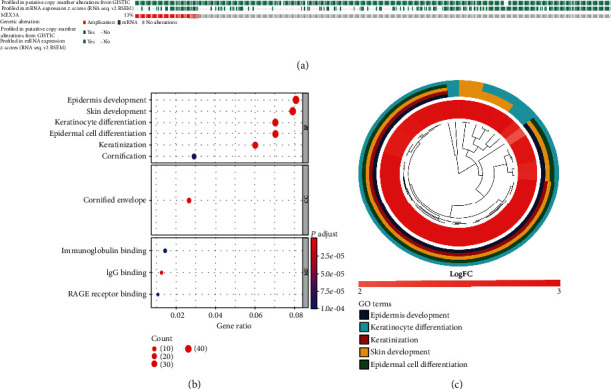
Analyses of genetic variations of MEX3A in OC (cBioPortal). (a) OncoPrint visual summary of variations on MEX3A. The different types of genetic alterations are represented by different colors. (b, c) Functional enrichment analyses of genes coexpressed with MEX3A.

Based on the above analysis, MEX3A is closely related to tumor immunology. In order to better understand the potential immune mechanism of MEX3A in cancer, genetic variations of MEX3A retrieved from the TCGA database (489 cases, Nature 2011) were analyzed by using the cBioPortal database. The results showed mRNA expression changes in 60 cases (16%), amplification in 38 cases (10%), a mutation in 1 case (0.3%), and multiple alterations in 19 cases (5%), in which amplification was the most common type (Figure 5(a)). Further, the expression of 771 genes was positively related to MEX3A and was increased with the amplification of MEX3A. Among these genes, LAMTOR2 had the most frequent alterations (Table 2). LAMTOR2 is essential for macrophage and dendritic cell (DC) homeostasis via mediating immune responses [19, 20]. Significantly enriched GO analysis showed that these genes encoded proteins that were mainly localized to the cornified envelope (Figures 5(b) and 5(c)). They were primarily involved in immunoglobulin binding, IgG binding, and RAGE reporter binding.

### 3.5. Construction of a Gene-Gene Interaction Network

To further explore the potential mechanism of MEX3A in promoting OC progression, we constructed a gene-gene interaction network by using the GeneMANIA database. Their functions were also analyzed. MEX3A were surrounded by 20 nodes representing genes that were greatly correlated with the family in terms of physical interactions, coexpression, prediction, colocalization, pathway, genetic interactions, and shared protein domains. From the results (Figure 3), we found that PABPC1, a kind of shuttling protein from the cytoplasm to nucleus in most eukaryotes, was correlated with MEX3A for physical interactions. PABPC1 is important for protein translation initiation and decay by binding to regulatory proteins [21]. In addition, KHDRBS2 was associated with IGF2BP2 and MEX3A in terms of shared protein domains. KHDRBS2 is also an RNA-binding protein that is tyrosine phosphorylated by Src during mitosis [22]. IGF2BP2 was colocalized with STRA6. Further functional analysis revealed that most proteins were greatly correlated with skeletal system development and genitalia development.

### 3.6. MEX3A Promoted Ovarian Cell Proliferation, Migration, and Invasion *In Vitro*

**Figure 6:**
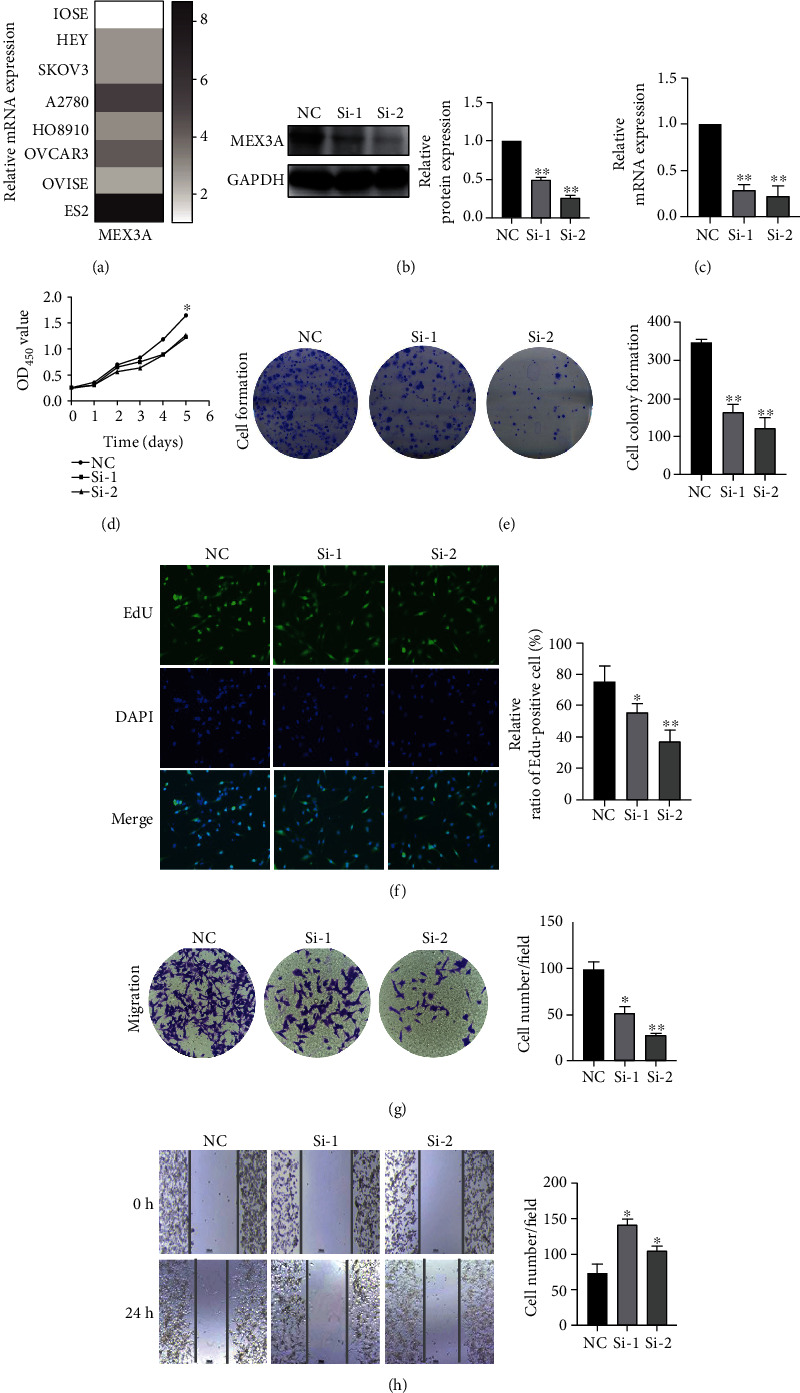
MEX3A promotes proliferation, migration, and invasion of ES2 cells in vitro. (a) Relative mRNA level of MEX3A in ovarian cancer cell lines. (b, c) The mRNA and protein expression of MEX3A in MEX3A knockdown ES2 cell treated with shRNA. (d, f) The CCK-8 assay, colony formation assay, and EdU assay showed that MEX3A knockdown in ES2 cells could suppress proliferative capability. (g, h) Migration and wound healing assay were utilized to evaluate and identify metastasis ability after MEX3A knockdown in ES2 cells. The data are presented as the mean ± SD (*n* = 3). ^∗^*P* < 0.05, ^∗∗^*P* < 0.01, and ^∗∗∗^*P* < 0.001.

To further evaluate the biological functions of MEX3A on ovarian cancer, the expression of MEX3A in different cell lines was tested, and *in vitro* studies were performed (Figure 6(a)). The ES2 cell line was chosen for further study. We silenced MEX3A expression by siRNA, and a nontargeting siRNA was used as a control. The efficiency was evaluated by Western blotting and RT-PCR (Figures 6(b) and 6(c)). We first studied its influence on OC growth by using CCK8 assay, clone formation assay, and EdU assay. Compared with the normal control group, MEX3A knockdown partly suppressed the proliferation of OC cells (*P* < 0.05, Figure 6(d)). Similarly, the colony number was significantly smaller than that of the control group (*P* < 0.05, Figure 6(e)). EdU is a thymidine nucleoside analogue, which is involved in DNA replication when targeting proliferating cells. The proliferation activity of ES2 cells can be analyzed with the number of red/blue fluorescence spots.  Figure 6(f) shows that compared with the control group, knockdown of MEX3A significantly inhibits the EdU uptake rate, which also indicates suppressed proliferation ability. Next, we assessed the role of MEX3A knockdown on the migration ability of OC. Transwell assay and wound healing assay were performed, and the results showed that MEX3A knockdown significantly inhibited cell migration in ES2 cells in comparison to the control group (Figures 6(g) and 6(h)). Collectively, these results indicated that MEX3A could promote the proliferation and migration in OC cells.

## 4. Discussion

OC is usually detected during the late stages; thus, few patients are eligible for timely treatment. Identifying sensitive and specific biomarkers for improving diagnosis and accurately evaluating prognosis continues to be an important research focus. In this study, we explored a novel gene—MEX3A—which is an RNA-binding protein or an E3 ubiquitin ligase acting posttranscriptional regulation, associated with the diagnosis and prognosis of OC. MEX3A has important roles in biological processes. Its expression is associated with intestinal homeostasis by regulating intestinal differentiation and promoting high expression of intestinal stem cell markers (LGR5, BMI1, and MSI1) [23, 24]. Moreover, a few studies have evaluated the effect of MEX3A on tumors. Abnormal activation of MEX3A can promote tumor cell proliferation, metastasis, and migration in gastric cancer and pancreatic ductal adenocarcinoma, breast cancer, and osteosarcoma [6, 25–27]. For example, MEX3A may act as a tumor promoter for breast cancer by regulating PIK3CA. Also, MEX3A could combine RIG-I to promote its ubiquitylation and proteasome-dependent degradation, which is beneficial for tumorigenesis [28].

However, the mechanisms of the MEX3A function have yet to be elucidated in OC. To the best of our knowledge, this is the first study that reported the role of MEX3A in OC through bioinformatics analysis of public sequencing data to guide future research in OC.

First, the results of the prognostic analysis showed that upregulation of MEX3A mRNA expression had the greatest correlation with poor OS and PFS in OC patients. In addition, we performed a series of in vitro experiments, which proved the inhibition of OC development by MEX3A knockdown. Hence, we speculate that MEX3A is extremely important as a prognostic indicator in OC patients and can be used as a predictor of tumor proliferation and metastasis. These results are consistent with bladder cancer, lung adenocarcinoma, and glioma [29–31]. Liang et al. found that MEX3A could enhance the instability of LAMA2 mRNA to promote lung adenocarcinoma metastasis by the PI3K/AKT pathway. In addition, they reported that MEX3A exerted its ubiquitination role to induce glioma tumorigenesis.

To explore the specific mechanisms of MEX3A in OC, a comprehensive bioinformatic analysis of MEX3A has been performed. Copy number variations (CNVs) have major genomic implications in human diseases, especially cancer, which can lead to phenotypic differences [30]. We found that the major CNV type of MEX3A was amplification, which was associated with shorter survival. Besides, neighboring gene networks close to MEX3A generally showed different degrees of amplification in OC. The genes coexpressed with MEX3A were subjected to functional and pathway enrichment analyses, and the results indicated that they were mainly involved in the immune response processes during tumorigenesis and progression of ovarian cancer.

We also constructed a gene-gene interaction network. The results suggested that MEX3A interacted intensively with other genes, such as PABPC1 and LAMTOR2. PABPC1 has been reported to bind the poly(A) tails of mRNAs, regulating the stability and biofunction of lncRNAs, which have critical roles in OC progression [31, 32]. PABPC1 could promote the binding of hnRNPLL (a plasma cell-specific RBP) to the immunoglobulin mRNA and regulate switching from mIgH to sIgH in plasma cells [33]. Yu et al. reported that PABPC1 could involve innate immune surveillance by regulating the activity of NK cells [34]. LAMTOR2, a regulator/LAMTOR complex member, activates AKT/mTOR to regulate dendritic cell homeostasis [20]. They implied that MEX3A might have an essential role in immunity by combining with PABPC1. Therefore, these results suggested that MEX3A and its related genes together regulate OC progression by a complex regulatory network.

Another important aspect of this study was that MEX3A expression was related to immune infiltration in OC. Our results demonstrated a moderate to a strong relationship between MEX3A expression level and infiltration level of macrophages, neutrophils, dendritic cells (DCs), B cells, and CD8+ T cells. Furthermore, immune cell activation and immunomodulators have been known for reducing mortality rates in patients with OC. In our study, we assessed the correlation between MEX3A and the immune system via the TIMER and TISIDB database, finding that MEX3A had the greatest correlation with lymphocytes (such as B cells, CD8+ T cells, neutrophils, and dendritic cells (DCs) and macrophages), immune inhibitors (such as IL10RB, IDO1, VTCN1, and HAVCR2), immunostimulators (such as C10orf54, TNFRSF18, TNFRSF14, and TNFRSF13C), and MHC molecules (such as B2M HLA-DMA HLA-DPA1, and HLA-DPB1). Therefore, MEX3A, which is associated with these immune-related genes, may provide a new target in immune therapy for OC.

The present study has several limitations. First, most results on the transcriptional level may reflect some aspects of immune infiltration. Also, reported findings need to be confirmed with larger clinical samples and experimental data using molecular biology techniques. Finally, we plan to further deepen our understanding of the underlying mechanism of immunomodulators related to MEX3A in our future work.

In conclusion, this study demonstrated that high MEX3A expression was correlated with poor prognosis and increased immune infiltration levels in macrophages, neutrophils, dendritic cells (DCs), B cells, and CD8+ T cells in OC. Our study provides a novel insight into the potential role of MEX3A as a cancer biomarker from the perspective of tumor immunology.

## Figures and Tables

**Table 1:** Survival analysis of MEX3A mRNA in multiple cancers.

Dataset		Endpoint	Probe ID	Number	Corrected *P* value	COX *P* value	In (HR) HR (95% CI-low CI-up)
GSE9891	Overall survival	226346_at	278	0.013633	0.007141	0.27	1.31 (1.08-1.60)
GSE9891	Overall survival	227512_at	278	0.114667	0.022613	0.25	1.28 (1.04-1.59)
GSE17260	Overall survival	A_24_P857404	110	0.004934	0.031824	-0.1.36	0.72 (0.53-0.97)
GSE17260	Overall survival	A_32_P96036	110	0.001922	0.097382	-1.23	0.78 (0.58-1.05)
GSE17260	Progression-free survival	A_32_P96036	110	0.044430	0.338936	-0.11	0.90 (0.72-1.12)

**Table 2:** The expression of 771 related genes with MEX3A in cBioPortal.

Gene	LogFC	entrezID
LAMTOR2	>10	28956
RAB25	>10	57111
UBQLN4	7.9	56893
LMNA	7.86	4000
SSR2	6.9	6746
ARHGEF2	6.31	9181
RXFP4	6.27	339403
KHDC4	5.86	22889
SEMA4A	7.73	64218
SLC25A44	7.68	9673
SCARNA4	6.19	677771
SNORA80E	6.19	677823
SYT11	6.14	23208
RIT1	5.73	6016
PMF1	6.64	11243
PMF1-BGLAP	6.64	100527963
GON4L	5.68	54856
BGLAP	6.48	632
PAQR6	5.9	79957
SMG5	5.9	23381
CCT3	5.84	7203
GLMP	5.84	112770
TMEM79	5.84	84283
VHLL	5.84	391104
ASH1L-AS1	4.95	645676
DAP3	4.95	7818
MSTO1	4.95	55154
YY1AP1	4.95	55249
MSTO2P	4.73	100129405
TSACC	5.73	128229
ASH1L	4.13	55870
C1ORF61	5.67	NA
RHBG	5.67	57127
POU5F1P4	4.16	645682
LRRC71	6.98	149499
TRIM46	4.2	80128
DPM3	4.14	54344
EFNA1	4.14	1942
GBA	4.14	2629
GBAP1	4.14	2630
KRTCAP2	4.14	200185
MTX1	4.14	4580
MUC1	4.14	4582
SLC50A1	4.14	55974
THBS3	4.14	7059
ARHGEF11	6.9	9826
HDGF	5.98	3068
INSRR	5.98	3645
ISG20L2	5.98	81875
MRPL24	5.98	79590
NTRK1	5.98	4914
PEAR1	5.98	375033
PRCC	5.98	5546
RRNAD1	5.98	51093
SH2D2A	5.98	9047
FAM189B	3.99	10712
SCAMP3	3.99	10067
CLK2	3.79	1196
FDPS	3.79	2224
HCN3	3.79	57657
PKLR	3.79	5313
RUSC1	3.79	23623
RUSC1-AS1	3.79	284618
IQGAP3	5.39	128239
MEF2D	5.39	4209
CRABP2	5.9	1382
CYCSP52	6.82	360155
ETV3	6.82	2117
ETV3L	6.82	440695
EFNA4	4.19	1945
ZBTB7B	4.19	51043
ADAM15	4.02	8751
DCST1	4.02	149095
DCST1-AS1	4.02	100505666
DCST2	4.02	127579
EFNA3	4.02	1944
BCAN	5.31	63827
GPATCH4	5.31	54865
HAPLN2	5.31	60484
NAXE	5.31	128240
NES	5.31	10763
TTC24	5.31	164118
FCRL4	6.64	83417
FCRL5	6.64	83416
CKS1B	4.05	1163
FLAD1	4.05	80308
LENEP	4.05	55891
ADAR	4.58	103
KCNN3	3.88	3782
PBXIP1	3.88	57326
PMVK	3.88	10654
PYGO2	3.88	90780
SHC1	3.88	6464
CD5L	6.54	922
FCRL1	6.54	115350
FCRL2	6.54	79368
FCRL3	6.54	115352
KIRREL1	6.54	55243
CHRNB2	4.49	1141
SHE	4.23	126669
TDRD10	4.23	126668
UBE2Q1	4.23	55585
CREB3L4	3.9	148327
CRTC2	3.9	200186
DENND4B	3.9	9909
JTB	3.9	10899
RAB13	3.9	5872
RPS27	3.9	6232
SLC39A1	3.9	27173
NUP210L	3.65	91181
CD1A	6.43	909
CD1B	6.43	910
CD1C	6.43	911
CD1D	6.43	912
CD1E	6.43	913
LINC01704	6.43	646268
OR10K2	6.43	391107
OR10T2	6.43	128360
IL6R	4.01	3570
C1ORF189	3.73	NA
C1ORF43	3.73	NA
HAX1	3.73	10456
TPM3	3.73	7170
UBAP2L	3.73	9898
OR10K1	5.43	391109
OR10R2	5.43	343406
GATAD2B	3.28	57459
SLC27A3	3.28	11000
PSMD4	2.95	5710
AQP10	3.65	89872
ATP8B2	3.65	57198
C2CD4D	3.05	100191040
C2CD4D-AS1	3.05	100132111
LINGO4	3.05	339398
MRPL9	3.05	65005
OAZ3	3.05	51686
RORC	3.05	6097
TDRKH	3.05	11022
THEM5	3.05	284486
INTS3	3.17	65123
SNAPIN	3.17	23557
LYSMD1	2.87	388695
PIP5K1A	2.87	8394
SCNM1	2.87	79005
TMOD4	2.87	29765
TNFAIP8L2	2.87	79626
TNFAIP8L2-SCNM1	2.87	100534012
VPS72	2.87	6944
MNDA	4.84	4332
LCE2B	3.49	26239
LCE2C	3.49	353140
LCE2D	3.49	353141
THEM4	2.96	117145
SEMA6C	2.8	10500
ILF2	3.07	3608
NPR1	3.07	4881
OR10X1	5.31	128367
OR10Z1	5.31	128368
OR6K6	5.31	128371
OR6N1	5.31	128372
OR6N2	5.31	81442
OR6P1	5.31	128366
OR6Y1	5.31	391112
TCHH	3.2	7062
TCHHL1	3.2	126637
CGN	2.88	57530
PLEKHO1	2.88	51177
VPS45	2.88	11311
ZNF687	2.88	57592
LCE3A	3.36	353142
LCE3B	3.36	353143
LCE3C	3.36	353144
LCE3D	3.36	84648
LCE3E	3.36	353145
HORMAD1	2.6	84072
CHTOP	2.98	26097
ANP32E	2.8	81611
APH1A	2.8	51107
C1ORF54	2.8	NA
CA14	2.8	23632
CIART	2.8	148523
MRPS21	2.8	54460
POGZ	2.8	23126
NBPF18P	3.09	441908
RPTN	3.09	126638
S100A10	3.09	6281
S100A11	3.09	6282
ANXA9	2.66	8416
MINDY1	2.66	55793
PRUNE1	2.66	58497
GOLPH3L	2.54	55204
CRCT1	3.23	54544
LCE5A	3.23	254910
CELF3	2.89	11189
RIIAD1	2.89	284485
S100A1	2.89	6271
S100A13	2.89	6284
S100A14	2.89	57402
TUFT1	2.89	7286
C1ORF68	3.41	NA
LCE2A	3.41	353139
LCE4A	3.41	199834
ACKR1	4.73	2532
AIM2	4.73	9447
CADM3	4.73	57863
CADM3-AS1	4.73	100131825
FCER1A	4.73	2205
IFI16	4.73	3428
OR10J3	4.73	441911
OR6K2	4.73	81448
OR6K3	4.73	391114
PYHIN1	4.73	149628
SPTA1	4.73	6708
GABPB2	2.6	126626
OTUD7B	2.81	56957
PI4KB	2.81	5298
PSMB4	2.81	5692
RFX5	2.81	5993
S100A2	2.81	6273
SELENBP1	2.81	8991
SNX27	2.81	81609
CRNN	3.12	49860
PRPF3	2.66	9129
ADAMTSL4	2.54	54507
ADAMTSL4-AS1	2.54	574406
MCL1	2.54	4170
KPRP	3.27	448834
LCE1F	3.27	353137
S100A16	2.73	140576
BNIPL	2.59	149428
C1ORF56	2.59	NA
CDC42SE1	2.59	56882
CERS2	2.59	29956
MLLT11	2.59	10962
FLG	3.01	2312
FLG-AS1	3.01	339400
FLG2	3.01	388698
CTSS	2.48	1520
ENSA	2.48	2029
LCE1A	3.14	353131
LCE1B	3.14	353132
LCE1C	3.14	353133
LCE1D	3.14	353134
LCE1E	3.14	353135
LCE6A	3.14	448835
SPRR2C	3.14	6702
MTMR11	2.81	10903
SF3B4	2.81	10262
ARNT	2.53	405
CTSK	2.53	1513
ECM1	2.53	1893
FALEC	2.53	100874054
RPRD2	2.53	23248
SETDB1	2.53	9869
TARS2	2.53	80222
HRNR	2.91	388697
S100A3	2.73	6274
S100A4	2.73	6275
S100A5	2.73	6276
S100A6	2.73	6277
OR10J1	4.6	26476
OR10J5	4.6	127385
LOC101928009	3.03	101928009
SMCP	3.03	4184
SPRR2G	3.03	6706
SV2A	2.58	9900
PGLYRP4	2.92	57115
S100A12	2.92	6283
S100A8	2.92	6279
S100A9	2.92	6280
SPRR2A	3.05	6700
SPRR2B	3.05	6701
SPRR2E	3.05	6704
SPRR2F	3.05	6705
APCS	4.19	325
S100A7	2.82	6278
S100A7A	2.82	338324
S100A7L2	2.82	645922
LELP1	2.93	149018
LOR	2.93	4014
PGLYRP3	2.93	114771
PRR9	2.93	574414
SPRR2D	2.93	6703
NOTCH2	2.44	4853
DUSP23	3.87	54935
LMOD1	3.51	25802
TIMM17A	3.51	10440
SPRR1B	2.83	6699
SPRR4	2.83	163778
SRGAP2D	2.28	100996712
IVL	2.73	3713
SPRR1A	2.73	6698
SPRR3	2.73	6707
LY9	4.05	4063
ACP6	2.42	51205
ANKRD20A12P	2.42	100874392
ANKRD34A	2.42	284615
ANKRD35	2.42	148741
BCL9	2.42	607
BOLA1	2.42	51027
CD160	2.42	11126
CHD1L	2.42	9557
EMBP1	2.42	647121
FAM72B	2.42	653820
FAM72D	2.42	728833
FCGR1A	2.42	2209
FCGR1B	2.42	2210
FCGR1CP	2.42	100132417
FMO5	2.42	2330
GJA5	2.42	2702
GJA8	2.42	2703
GNRHR2	2.42	114814
GPR89A	2.42	653519
GPR89B	2.42	51463
HIST2H2AA3	2.42	8337
HIST2H2AB	2.42	317772
HIST2H2AC	2.42	8338
HIST2H2BA	2.42	337875
HIST2H2BC	2.42	337873
HIST2H2BE	2.42	8349
HIST2H2BF	2.42	440689
HIST2H3A	2.42	333932
HIST2H3D	2.42	653604
HIST2H4A	2.42	8370
HJV	2.42	148738
ITGA10	2.42	8515
LINC00623	2.42	728855
LINC00624	2.42	100289211
LINC00869	2.42	57234
LINC01138	2.42	388685
LINC02591	2.42	388692
LIX1L	2.42	128077
LOC102723769	2.42	102723769
LOC653513	2.42	653513
LOC728989	2.42	728989
LSP1P5	2.42	645166
NBPF10	2.42	100132406
NBPF11	2.42	200030
NBPF12	2.42	149013
NBPF13P	2.42	644861
NBPF14	2.42	25832
NBPF15	2.42	284565
NBPF17P	2.42	401967
NBPF20	2.42	100288142
NBPF25P	2.42	101929780
NBPF8	2.42	728841
NBPF9	2.42	400818
NOTCH2NLA	2.42	388677
NUDT17	2.42	200035
PDE4DIP	2.42	9659
PDIA3P1	2.42	171423
PDZK1	2.42	5174
PDZK1P1	2.42	100034743
PEX11B	2.42	8799
PFN1P2	2.42	767846
PIAS3	2.42	10401
POLR3C	2.42	10623
POLR3GL	2.42	84265
PPIAL4A	2.42	653505
PPIAL4D	2.42	645142
PPIAL4E	2.42	730262
PPIAL4G	2.42	644591
PRKAB2	2.42	5565
RBM8A	2.42	9939
RNF115	2.42	27246
SEC22B	2.42	9554
SRGAP2-AS1	2.42	100873165
TXNIP	2.42	10628
ELF3	3.31	1999
PIK3C2B	3.31	5287
CFAP45	3.6	25790
CRP	3.6	1401
FCRL6	3.6	343413
IGSF9	3.6	57549
SLAMF8	3.6	56833
SLAMF9	3.6	89886
SNHG28	3.6	284677
TAGLN2	3.6	8407
VSIG8	3.6	391123
FAM72C	2.12	554282
ADAMTS4	4.31	9507
APOA2	4.31	336
ARHGAP30	4.31	257106
B4GALT3	4.31	8703
CFAP126	4.31	257177
DEDD	4.31	9191
DUSP12	4.31	11266
F11R	4.31	50848
FCER1G	4.31	2207
FCGR2A	4.31	2212
FCGR2B	4.31	2213
FCGR2C	4.31	9103
FCGR3A	4.31	2214
FCGR3B	4.31	2215
FCRLA	4.31	84824
FCRLB	4.31	127943
HSPA6	4.31	3310
HSPA7	4.31	3311
ITLN2	4.31	142683
KLHDC9	4.31	126823
LOC101928372	4.31	101928372
MPZ	4.31	4359
NDUFS2	4.31	4720
NECTIN4	4.31	81607
NIT1	4.31	4817
NR1I3	4.31	9970
PCP4L1	4.31	654790
PFDN2	4.31	5202
PPOX	4.31	5498
RPL31P11	4.31	641311
SDHC	4.31	6391
TOMM40L	4.31	84134
TSTD1	4.31	100131187
UFC1	4.31	51506
USF1	4.31	7391
USP21	4.31	27005
ATP1A2	3.14	477
RNPEP	3.14	6051
LINC01133	3.38	100505633
PPP1R15B	3.38	84919
CD48	3.73	962
SLAMF7	3.73	57823
ATP1A4	3.19	480
IGSF8	3.19	93185
KCNJ10	3.19	3766
KCNJ9	3.19	3765
MDM4	3.19	4194
PIGM	3.19	93183
ATF6	3.9	22926
CD244	3.9	51744
ITLN1	3.9	55600
LOC101928404	3.9	101928404
OLFML2B	3.9	25903
RGS4	3.9	5999
RGS5	3.9	8490
SHISA4	3.47	149345
SLAMF1	3.47	6504
LINC01142	4.14	284688
METTL11B	4.14	149281
CASQ1	3.24	844
CENPL	3.24	91687
DARS2	3.24	55157
DCAF8	3.24	50717
GAS5	3.24	60674
GAS5-AS1	3.24	100506046
IPO9	3.24	55705
LINC00628	3.24	127841
PEA15	3.24	8682
RC3H1	3.24	149041
SERPINC1	3.24	462
SNORD44	3.24	26806
SNORD47	3.24	26802
SNORD74	3.24	619498
SNORD75	3.24	692195
SNORD76	3.24	692196
SNORD77	3.24	692197
SNORD78	3.24	692198
SNORD79	3.24	26770
SNORD80	3.24	26774
SNORD81	3.24	26769
ZBTB37	3.24	84614
C1ORF226	3.58	NA
CCDC190	3.58	339512
LOC100422212	3.58	100422212
NUF2	3.58	83540
SH2D1B	3.58	117157
SPATA46	3.58	284680
UAP1	3.58	6675
UHMK1	3.58	127933
LAMC2	2.52	3918
NMNAT2	2.52	23057
ASTN1	3.05	460
BLACAT1	3.05	101669762
FLJ31356	3.05	403150
CD84	3.31	8832
DDR2	3.31	4921
HSD17B7	3.31	51478
LRRN2	3.31	10446
NOS1AP	3.31	9722
SLAMF6	3.31	114836
FMO2	3.73	2327
FMO3	3.73	2328
FMO6P	3.73	388714
GORAB	3.73	92344
KIFAP3	3.73	22920
MROH9	3.73	80133
PRRX1	3.73	5396
ANGPTL1	2.73	9068
FAM20B	2.73	9917
LAMC1	2.73	3915
RALGPS2	2.73	55103
TOR3A	2.73	64222
ETNK2	2.88	55224
FOSL2	2.88	2355
GOLT1A	2.88	127845
IPO9-AS1	2.88	100873949
KISS1	2.88	3814
LGR6	2.88	59352
NFASC	2.88	23114
PLEKHA6	2.88	22874
PTPRVP	2.88	148713
REN	2.88	5972
SNRPE	2.88	6635
SOX13	2.88	9580
ZC3H11A	2.88	9877
ABL2	2.6	27
PLB1	2.6	151056
CNTN2	3.09	6900
DSTYK	3.09	25778
KLHDC8A	3.09	55220
KLHL20	3.09	27252
NUAK2	3.09	81788
RBBP5	3.09	5929
TMEM81	3.09	388730
ATP1B1	3.95	481
BLZF1	3.95	8548
C1ORF112	3.95	NA
CCDC181	3.95	57821
LINC00626	3.95	79100
LINC00970	3.95	101978719
METTL18	3.95	92342
NME7	3.95	29922
SCYL3	3.95	57147
SELE	3.95	6401
SELL	3.95	6402
SELP	3.95	6403
SLC19A2	3.95	10560
ARL8A	2.73	127829
C1ORF21	2.73	NA
C1ORF220	2.73	NA
CSRP1	2.73	1465
GLUL	2.73	2752
GPR37L1	2.73	9283
LINC00272	2.73	388719
LINC00303	2.73	284573
LINC01344	2.73	400799
NAV1	2.73	89796
PTPN7	2.73	5778
RPS10P7	2.73	376693
SOAT1	2.73	6646
TEDDM1	2.73	127670
TEX35	2.73	84066
ZNF648	2.73	127665
NUCKS1	2.9	64710
OCLM	2.9	10896
ODR4	2.9	54953
PBX1	2.9	5087
PDC	2.9	5132
PRG4	2.9	10216
RAB29	2.9	8934
RNU6-72P	2.9	100873775
SLC41A1	2.9	254428
TPR	2.9	7175
ARPC5	2.59	10092
LINC01686	2.59	284648
NPL	2.59	80896
PHLDA3	2.59	23612
RABGAP1L	2.59	9910
RASAL2	2.59	9462
RGS16	2.59	6004
RGS8	2.59	85397
RGSL1	2.59	353299
RNASEL	2.59	6041
TSEN15	2.59	116461
FMO1	3.14	2326
FMO4	3.14	2329
LMX1A	3.14	4009
TOP1P1	3.14	7151
ANKRD36BP1	3.54	84832
CD247	3.54	919
CREG1	3.54	8804
DPT	3.54	1805
DUSP27	3.54	92235
F5	3.54	2153
FAM78B	3.54	149297
FMO9P	3.54	116123
GPA33	3.54	10223
ILDR2	3.54	387597
LINC01363	3.54	101928484
LOC100505918	3.54	100505918
MAEL	3.54	84944
POGK	3.54	57645
POU2F1	3.54	5451
SFT2D2	3.54	375035
TADA1	3.54	117143
TBX19	3.54	9095
TIPRL	3.54	261726
XCL1	3.54	6375
XCL2	3.54	6846
BRINP2	2.73	57795
CRYZL2P	2.73	730102
GS1-279B7.1	2.73	100288079
IVNS1ABP	2.73	10625
LINC01741	2.73	101928778
RASAL2-AS1	2.73	100302401
RNU6-79P	2.73	100873779
SWT1	2.73	54823
TRMT1L	2.73	81627
ZBED6	2.73	100381270
APOBEC4	2.47	403314
DHX9	2.47	1660
LHX4	2.47	89884
LHX4-AS1	2.47	100527964
SHCBP1L	2.47	81626
ERO1B	1.97	56605
ANKRD45	2.92	339416
BRINP3	2.92	339479
COPA	2.92	1314
EEF1AKNMT	2.92	51603
GPR52	2.92	9293
LEMD1	2.92	93273
LEMD1-AS1	2.92	284576
LINC01720	2.92	440704
LOC100505716	2.92	100505716
MYOC	2.92	4653
NCSTN	2.92	23385
NHLH1	2.92	4807
PEX19	2.92	5824
PLA2G4A	2.92	5321
PRRC2C	2.92	23215
RXRG	2.92	6258
SCARNA3	2.92	677679
SUMO1P3	2.92	474338
TEX50	2.92	730159
TMCC2	2.92	9911
VAMP4	2.92	8674
VANGL2	2.92	57216
ADORA1	2.58	134
AXDND1	2.58	126859
CHI3L1	2.58	1116
CHIT1	2.58	1118
FAM163A	2.58	148753
LAX1	2.58	54900
LINC01350	2.58	101929093
MYBPH	2.58	4608
MYOG	2.58	4656
NIBAN1	2.58	116496
NPHS2	2.58	7827
PM20D1	2.58	148811
PPFIA4	2.58	8497
PPP1CB	2.58	5500
RNF2	2.58	6045
SEC16B	2.58	89866
TDRD5	2.58	163589
TOR1AIP1	2.58	26092
TOR1AIP2	2.58	163590
COLGALT2	2.35	23127
RGL1	2.35	23179
SMG7-AS1	2.35	284649
ADCY10	3.21	55811
GPR161	3.21	23432
MPC2	3.21	25874
MPZL1	3.21	9019
RCSD1	3.21	92241
SLC4A1AP	3.21	22950
SUPT7L	3.21	9913
SUGCT	>10	79783
MTR	1.99	4548
C1ORF53	2.73	NA
CDK18	2.73	5129
COP1	2.73	64326
LHX9	2.73	56956
LOC100505795	2.73	100505795
NEK7	2.73	140609
PPP1R12B	2.73	4660
PTGS2	2.73	5743
SLC9C2	2.73	284525
NCF2	2.24	4688
PLEKHG2	1.56	64857
ZFP36	1.56	7538
BTG2	2.44	7832
CACNA1E	2.44	777
CEP350	2.44	9857
CTSE	2.44	1510
EDEM3	2.44	80267
FLJ23867	2.44	NA
FMOD	2.44	2331
HMCN1	2.44	83872
LINC01136	2.44	730227
LINC01699	2.44	100287948
PAPPA2	2.44	60676
QSOX1	2.44	5768
TNNI1	2.44	7135
MT1HL1	1.92	645745
LRRC52	2.95	440699
MRPL33	2.95	9553
TNFSF18	2.95	8995
GMFG	1.52	9535
MED29	1.52	55588
PAF1	1.52	54623
SAMD4B	1.52	55095
SMG7	2.14	9887
ACTN2	1.85	88
EDARADD	1.85	128178
GPR137B	1.85	7107
HEATR1	1.85	55127
LGALS8	1.85	3964
LGALS8-AS1	1.85	100287902
NID1	1.85	4811
RYR2	1.85	6262
DENND1B	2.56	163486
GLRX2	2.56	51022
RO60	2.56	6738
SLC45A3	2.56	85414
SRGAP2	2.56	23380
SRGAP2B	2.56	647135
SRGAP2C	2.56	653464
SUCO	2.56	51430
UBE2T	2.56	29089
DNM3	2.31	26052
IER5	2.31	51278
KIAA1614	2.31	57710
LINC00260	2.31	84719
LOC284581	2.31	284581
MR1	2.31	3140
PAX8	2.31	7849
PAX8-AS1	2.31	654433
SNORA77	2.31	677843
STX6	2.31	10228
LRATD1	4.05	151354
C19ORF47	1.94	NA
ARID4B	1.79	51742
CHRM3	1.79	1131
CHRM3-AS1	1.79	100873984
GGPS1	1.79	9453
TBCE	1.79	6905
KLRF2	2.05	100431172
LINC01132	1.67	100506810
ALDH9A1	2.73	223
DCAF6	2.73	55827
KIAA0040	2.73	9674
LINC01351	2.73	101929120
LOC440700	2.73	440700
LRRC52-AS1	2.73	400794
MGST3	2.73	4259
RBKS	2.73	64080
TNFSF4	2.73	7292
TNR	2.73	7143
UCK2	2.73	7371
ATP2B4	2.2	493
RHEX	2.2	440712
SLC26A9	2.2	115019
B3GALT2	2.41	8707
BABAM2	2.41	9577
CDC73	2.41	79577
CRB1	2.41	23418
ELK4	2.41	2005
FAM72A	2.41	729533
MFSD4A	2.41	148808
MFSD4A-AS1	2.41	284578
PHF12	2.41	57649
PIGC	2.41	5279
RGS1	2.41	5996
RGS13	2.41	6003
RGS2	2.41	5997
TMEM183A	2.41	92703
TMEM183B	2.41	653659
UCHL5	2.41	51377
AGT	1.87	183
C1ORF198	1.87	NA
CAPN9	1.87	10753
COG2	1.87	22796
ZP4	1.87	57829
DYRK1B	1.47	9149
FBL	1.47	2091
IFNL2	1.47	282616
IFNL3	1.47	282617
IFNL4	1.47	101180976
NCCRP1	1.47	342897
RPS16	1.47	6217
SYCN	1.47	342898

## Data Availability

Previously reported [RNA-Seq] TCGA data were used to support this study and are available at GEPIA (doi: 10.1093/nar/gkx247), cBioPortal (doi: 10.1126/scisignal.2004088), LinkedOmics (doi: 10.1093/nar/gkx1090), and the Kaplan–Meier plotter (doi: 10.1530/ERC-11-0329). These prior studies (and datasets) are cited at relevant places within the text as references [9–11, 15]. There is no research data used to support this study.

## Supplementary Materials

Supplementary Materials Figure S1: the mRNA expression levels of MEX3A in various cancers and prognostic values of MEX3A in ovarian cancer in the Kaplan–Meier plotter. (a) The expression of MEX3A in various cancers from Oncomine. The threshold was designed with the following parameters: fold change = 2 and *P* value = 0.01. The color intensity (red or blue) is directly proportional to the significance level of upregulation or downregulation. (b) The MEX3A expression levels in different tumor types from the TCGA database were determined by GEPIA. (c, d) Prognostic significance of MEX3A in serous ovarian carcinoma. (e, f) Prognostic significance of MEX3A in endometrioid carcinoma. ^∗^*P* < 0.05, ^∗∗^*P* < 0.01, and ^∗∗∗^*P* < 0.001.
