# Supplementary material for: Comprehensive Analysis of Prognostic Value of MEX3A and Its Relationship with Immune Infiltrates in Ovarian Cancer
Source: J Immunol Res. 2021 Jun 3;2021:5574176. doi: 10.1155/2021/5574176 (PMC8195639; doi:10.1155/2021/5574176)
Supplement: Supplementary Materials — Figure S1: the mRNA expression levels of MEX3A in various cancers and prognostic values of MEX3A in ovarian cancer in the Kaplan–Meier plotter. (a) The expression of MEX3A in various cancers from Oncomine. The threshold was designed with the following parameters: fold change = 2 and P value = 0.01. The color intensity (red or blue) is directly proportional to the significance level of upregulation or downregulation. (b) The MEX3A expression levels in different tumor types from the TCGA database were determined by GEPIA. (c, d) Prognostic significance of MEX3A in serous ovarian carcinoma. (e, f) Prognostic significance of MEX3A in endometrioid carcinoma. ∗P < 0.05, ∗∗P < 0.01, and ∗∗∗P < 0.001. [file 5574176.f1.zip › 5574176.f1/sup Figure 1.pdf]

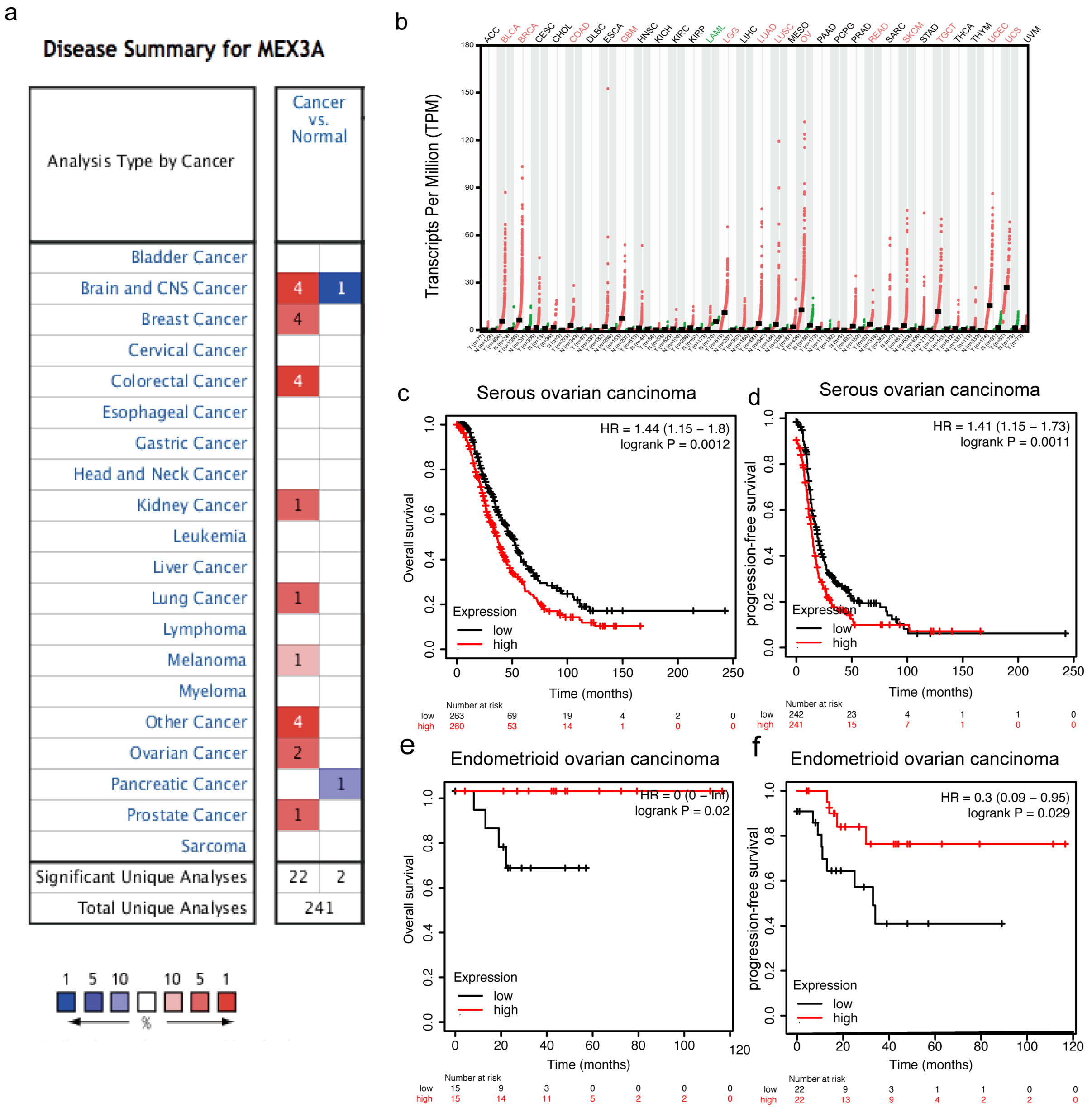
a
Disease Summary for MEX3A
Analysis Type by Cancer
Cancer vs. Normal
Bladder Cancer
Brain and CNS Cancer 4 1
Breast Cancer 4
Cervical Cancer
Colorectal Cancer 4
Esophageal Cancer
Gastric Cancer
Head and Neck Cancer
Kidney Cancer 1
Leukemia
Liver Cancer
Lung Cancer 1
Lymphoma
Melanoma 1
Myeloma
Other Cancer 4
Ovarian Cancer 2
Pancreatic Cancer 1
Prostate Cancer 1
Sarcoma
Significant Unique Analyses 22 2
Total Unique Analyses 241
1 5 10 10 5 1
%
b
ACC BLCA BRCA CESC CHOL COAD DLBC ESCA GBM HNSC KICH KIRC KIRP LAML LGG LIHC LUAD LUSC MESO OV PAAD PCPG PRAD READ SARC SKCM STAD TGCT THCA THYM UCEC UCS UVM
Transcripts Per Million (TPM)
180
150
120
90
60
30
0
c
Serous ovarian cancinoma
HR = 1.44 (1.15 – 1.8)
logrank P = 0.0012
Overall survival
Expression
low
high
Time (months)
Number at risk
low 263 69 19 4 2 0
high 260 53 14 1 0 0
d
Serous ovarian cancinoma
HR = 1.41 (1.15 – 1.73)
logrank P = 0.0011
progression-free survival
Expression
low
high
Time (months)
Number at risk
low 242 23 4 1 1 0
high 241 15 7 1 0 0
e
Endometrioid ovarian cancinoma
HR = 0 (0 – Inf)
logrank P = 0.02
Overall survival
Expression
low
high
Time (months)
Number at risk
low 15 9 3 0 0 0 0
high 15 14 11 5 2 2 0
f
Endometrioid ovarian cancinoma
HR = 0.3 (0.09 – 0.95)
logrank P = 0.029
progression-free survival
Expression
low
high
Time (months)
Number at risk
low 22 9 3 1 1 0 0
high 22 13 9 4 2 2 0
