# Supplementary figures and images for: Comprehensive Analysis of Prognostic Value of MEX3A and Its Relationship with Immune Infiltrates in Ovarian Cancer
Source: J Immunol Res. 2021 Jun 3;2021:5574176. doi: 10.1155/2021/5574176 (PMC8195639; doi:10.1155/2021/5574176)

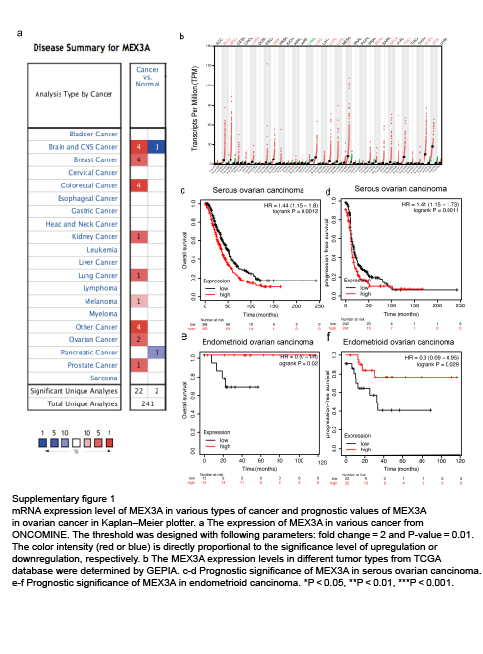

Supplement: Supplementary Materials — Figure S1: the mRNA expression levels of MEX3A in various cancers and prognostic values of MEX3A in ovarian cancer in the Kaplan–Meier plotter. (a) The expression of MEX3A in various cancers from Oncomine. The threshold was designed with the following parameters: fold change = 2 and P value = 0.01. The color intensity (red or blue) is directly proportional to the significance level of upregulation or downregulation. (b) The MEX3A expression levels in different tumor types from the TCGA database were determined by GEPIA. (c, d) Prognostic significance of MEX3A in serous ovarian carcinoma. (e, f) Prognostic significance of MEX3A in endometrioid carcinoma. ∗P < 0.05, ∗∗P < 0.01, and ∗∗∗P < 0.001. [file 5574176.f1.zip › 5574176.f1/Sup Figure 1.jpg]
